# Chemoradiation followed by adjuvant durvalumab in stage III non–small cell lung cancer: Real‐world comparison of treatment outcomes to historical controls treated with chemoradiation alone

**DOI:** 10.1111/1759-7714.14452

**Published:** 2022-05-11

**Authors:** Akram Saad, Jeffrey Goldstein, Sarit Appel, Sameh Daher, Damien Urban, Amir Onn, Hadas Gantz‐Sorotsky, Anastasiya Lobachov, Teodor Gottfried, Benjamin Spieler, Jair Bar

**Affiliations:** ^1^ Oncology Institute, Sheba Medical Center Tel‐Hashomer Israel; ^2^ Sackler Faculty of Medicine, Tel‐Aviv University Tel‐Aviv Israel; ^3^ Department of Radiation Oncology Tel‐Aviv Sourasky Medical Center Tel‐Aviv Israel; ^4^ Department of Radiation Oncology University of Miami School of Medicine Miami Florida USA

**Keywords:** durvalumab, immunotherapy, non–small cell lung cancer, PD‐L1, stage III

## Abstract

**Objective:**

Compare outcomes in patients with stage III non–small cell lung cancer (NSCLC) treated with chemoradiation and adjuvant durvalumab to historical controls treated with chemoradiation alone.

**Methods:**

The records of patients with stage III NSCLC treated with definitive chemoradiation ± adjuvant durvalumab were reviewed retrospectively. Primary endpoints were progression free survival (PFS), overall survival (OS), and adverse events (AE).

**Results:**

Between September 2009 and September 2020, 215 patients were treated with concurrent chemoradiation *(n* = 144) or concurrent chemoradiation followed by adjuvant durvalumab (*n* = 71). Compared to historical controls, durvalumab use was associated with improved PFS: median (27 months vs. 10 months, *p* < 0.0001), 1‐year (83.1% vs. 43.8, *p* < 0.0001); and improved OS; median (not reached vs. 24 months, *p* < 0.0001), 1‐year (85.9% vs. 81.9%, *p* < 0.0001). Multivariate analysis showed adjuvant durvalumab was associated with increased OS (*p* = 0.005) and PFS (*p* = 0.001). Within the durvalumab group, only clinical stage IIIA versus IIIB/C was associated with improved OS (*p* = 0.049), but not PFS. There was no association between PFS or OS and Eastern Cooperative Oncology Group (ECOG) score, prior history of immune disease, programmed death‐ligand 1 (PD‐L1) receptor status, delay in starting durvalumab beyond 42 days, or development of an AE. During durvalumab treatment, 63 AE were reported in 52 patients with treatment discontinuation in 11. Pneumonitis was the most common AE reported (*n* = 35, 49%). Most AE were grade 1–2 (*n* = 57). Grade 3–4 AE were uncommon (*n* = 6) and none were grade 5.

**Conclusion:**

Treatment with adjuvant durvalumab following chemoradiation was associated with improved PFS and OS compared to chemoradiation alone.

## Graphical abstract

Treatment of stage III NSCLC with adjuvant durvalumab was associated with improved PFS and OS

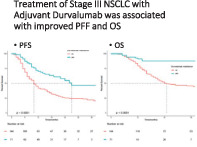

## INTRODUCTION

Most patients who develop non–small cell lung cancer (NSCLC) present with locally advanced or metastatic disease.^1^ When locally advanced disease is unresectable or patients are unfit to undergo resection, optimal treatment consists of concomitant chemoradiation using platinum‐based doublet chemotherapy and radiation to 60 Gray (Gy).^2^ ^,^ ^3^ ^,^ ^4^ Outcomes with concomitant chemoradiation alone are poor, with only 15%–30% of patients surviving 5 years post‐treatment.^2^, ^4^ The use of adjuvant chemotherapy following chemoradiation failed to improve these poor outcomes.^5^

The use of immunotherapy in patients with stage IV NSCLC is associated with improved overall survival (OS) and progression free survival (PFS).^6^ The PACIFIC trial reported improved outcomes in carefully selected patients with unresectable stage III NSCLC treated with 1 year of adjuvant durvalumab following chemoradiation compared to a control group treated with chemoradiation followed by placebo.^7^, ^8^, ^9^, ^10^, ^11^ Subsequent reports from real‐world single and multicenter studies confirmed the improved OS and PFS reported in the PACIFIC trial.^12^, ^13^, ^14^, ^15^, ^16^, ^17^, ^18^ As a result, consolidation of treatment with 1 year of adjuvant durvalumab has become the new standard of care for stage III unresectable NSCLC.^19^, ^20^ Since the publication of the PACIFIC trial, our institution offered treatment with adjuvant durvalumab to all patients with unresectable stage III NSCLC without disease progression following chemoradiation.

In this study, we compare outcomes in patients with unresectable stage III NSCLC treated with chemoradiation and adjuvant durvalumab to a cohort of historical controls treated with chemoradiation alone. We hypothesized that our cohort that included many patients that would have been excluded from the PACIFIC trial because of initiation of durvalumab therapy beyond 42 days, a history of prior autoimmune disease, or East Coast Oncology Group (ECOG) performance scores ≥2 would have similar outcomes to the results reported in the PACIFIC trial and have improved outcomes compared to the historical controls treated with chemoradiation alone.

Advantages of a single institution retrospective analysis include use of uniform criteria for staging, selection of treatment protocols, and chemoradiation delivery allowing for evaluation of effects of adjuvant durvalumab therapy on PFS, OS, and toxicity without confounding effects of treatment variation from different institutional approaches.

Review of anecdotal data from real‐world situations not encountered in clinical trials is useful for hypothesis generation and formulation of future trials and provides additional data for guiding clinicians in real‐world settings. Evaluation of safety and survival outcomes for patients treated with durvalumab that would have been excluded from the Pacific trial tests reproducibility and provides confirmation of results from previous controlled trials.

## METHODS

### Patients

Patient records from a tertiary academic institution lung cancer working database were reviewed retrospectively to compare outcomes in patients with stage III NSCLC treated with definitive chemoradiation with or without adjuvant durvalumab. The database was searched for all patients diagnosed with unresectable stage III NSCLC treated with definitive chemoradiation. Staging results done using computed tomography (CT), fluorodeoxyglucose (FDG)‐positron emission tomography (PET)/CT, contrast enhancing brain magnetic resonance imaging (MRI) and mediastinal staging were recorded. Performance status was recorded by the treating physicians using the ECOG scale. Programmed death‐ligand 1 (PD‐L1) expression was recorded if tested on archived tumor samples, measured by immunohistochemistry (Ventana Medical Systems) and grouped for analysis as follows: < 1%, 1%–49%, and ≥50%.

### Treatment

Patients were presented in a multidisciplinary tumor board for treatment recommendations. Patients with stage III NSCLC were offered definitive chemoradiation. After chemoradiation, patients' disease was reassessed for response and metastasis and if medically fit and the tumor resectable, were offered surgery. Patients completing chemoradiation without evidence of progressive disease at first post‐treatment imaging test who did not receive surgery were analyzed to serve as historical controls. Starting September 2017, all patients with unresectable disease or who were unfit for surgery were offered adjuvant durvalumab (10 mg/kg) every 2 weeks for 1 year. During the period from September 2017 to January 2019, the durvalumab was granted by AstraZeneca (Cambridge, UK) for compassionate use and afterward by insurance providers. No other immune check point inhibitors other than durvalumab were considered for use in this study.

The characteristics of the durvalumab‐treated group were analyzed independently and compared to the historical controls with respect to age, gender, smoking history, ECOG score, histology, stage, presence of biomarkers, PD‐L1 status, chemotherapy protocol, radiation dose and technique, OS, PFS, and adverse events (AE).

### Patient assessment

Institutional standard procedure indicated patients to be assessed weekly during chemoradiation and monthly during adjuvant durvalumab treatment. CT imaging of the chest was scheduled at 3‐month intervals for the first 2 years and then at 6‐month intervals thereafter. Additional imaging was performed based on patient symptoms or disease progression. PFS and OS were recorded from the last day of chemoradiation therapy to date of last follow‐up, progressive disease (PD), or death. Immune‐related AE after completion of chemoradiation were attributed to adjuvant durvalumab and were categorized and retrospectively recorded according to Common Terminology Criteria for Adverse Events (CTCAE) version 5.0.^21^ Cause for treatment interruptions or discontinuations were recorded as AE, PD, coronavirus disease (COVID)‐19 pandemic, or death.

### Statistics

The Kaplan–Meier (KM) method was used to estimate PFS and OS. χ^2^ test and Cox univariate (UVA) were performed to test significance of factors considered potentially important, for categorical and continuous variables, respectively. Variables with *p* values ≤0.1 on UVA were selected for inclusion in the multivariate analysis (MVA). R Core Team software was used for all statistical calculations (R Foundation for Statistical Computing; https://www.R-project.org/).

### Ethics

Data collection for this study was approved by the institutional ethics board for retrospective analysis (SMC‐2678‐15).

## RESULTS

### Patients

Between September 2009 and September 2020, 421 patients with stage III NSCLC were treated with chemoradiation and assessed for trial eligibility. Patients who received post‐operative chemoradiation following surgery for pathologic stage III disease (*n* = 77, 18.2%), underwent surgery after chemoradiation (*n* = 73, 17.3%) or who had PD at first evaluation after completing chemoradiation (*n* = 56, 13.3%) were excluded from analysis. The remaining 215 patients (51.1%) were treated with either concurrent chemoradiation alone (*n* = 144, 34%) or after September 2017, concurrent chemoradiation followed by adjuvant durvalumab (*n* = 71, 16.9%). Almost all patients eligible for treatment with adjuvant durvalumab received therapy (*n* = 77). Only six patients refused treatment and were included in the historical controls for analysis. No other immune checkpoint inhibitors were used in this study.

Data from our lung cancer registry show that during the entire study interval, 73 patients (17.3%) underwent surgery following definitive chemoradiation. Most of these patients (*n* = 67, 15.9%) underwent surgery before or soon after the introduction of durvalumab in 2017. Since 2019, the number of patients undergoing surgical resection following definitive chemoradiation decreased (*n* = 6, 1.4%).

Table 1 compares patient and tumor characteristics, treatment parameters, median duration of follow‐up, PFS, and OS for the durvalumab group with the historical controls. Median duration of follow‐up was 28.6 months (range, 3.8–116.9) for historical controls and 19.4 months (range, 6.0–40.7) for the durvalumab group. Compared to the historical controls, the durvalumab cohort was slightly older (*p* = 0.042), had an increased incidence of stage IIIA disease (*p* = 0.013), known PD‐L1 status (72% vs. 28%, *p* = 0.0596), and use of brain MRI for staging (94% vs. 65%, *p* = 0.001). History of prior autoimmune disease was uncommon in both groups. The groups were balanced with respect to gender, marital status, ECOG performance status, smoking status, weight loss, and histology. PET/CT imaging was used for staging in all patients in both groups, whereas mediastinoscopy for staging was used infrequently in both groups.

**TABLE 1:** Patient characteristics

Baseline characteristics	Chemoradiation followed by durvalumab (*n* = 71)	Chemoradiation alone (*n* = 144)	*p*‐value
Age, (years)			
Median age (range)	67 (40–82)	64 (45–81)	0.042
Gender			
Male	45 (63.4)	107 (74.3)	0.112
Female	26 (36.6)	37 (25.7)	
Marital status			
Single	23 (32.4)	43 (29.9)	0.754
Married	48 (67.6)	101 (70.1)	
Performance status			
ECOG 0	34 (47.9)	58 (40.3)	0.519
ECOG 1	33 (46.5)	74 (51.4)	
ECOG ≥2	4 (5.6)	12 (8.3)	
Smoking status			
Ever smoker	64 (90.1)	130 (90.3)	1
Never smoker	7 (9.6)	14 (9.7)	
Weight loss			
Yes (>5%)	43 (60.6)	97 (67.4)	0.362
No (<5%)	28 (39.4)	47 (32.7)	
Histology			
Adenocarcinoma	39 (54.9)	69 (47.9)	0.479
Squamous cell carcinoma	23 (32.4)	48 (33.3)	
NOS	9 (12.7)	27 (18.8)	
Stage			
IIIA	42 (59.2)	58 (40.3)	0.013
IIIB‐C	29 (40.8)	86 (59.7)	
Prior autoimmune disease			
Yes	3 (4.2)	4 (2.8)	0.1865
No	68 (95.8)	140 (97.2)	
PDL1 status (3 groups)			
≥50%	20 (28.2)	10 (6.9)	0.0596
≥1–49%	9 (12.7)	16 (11.1)	
<1%	22 (31.0)	14 (9.7)	
Unknown	20 (28.2)	104 (72.2)	
PDL1 status (2 groups)			
≥1%	29 (40.8)	26 (18.1)	0.5187
<1%	22 (31.0)	14 (9.7)	
Unknown	20 (28.2)	104 (72.2)	
Chemotherapy			
Carboplatin + paclitaxel	54 (76.1)	91 (63.2)	<0.0001
Cisplatin + etoposide	14 (19.7)	9 (6.3)	
Other	3 (4.2)	44 (30.6)	
Mode of chemoradiation			
Sequential	0	9 (6.3)	0.032
Concomitant	71 (100)	135 (93.8)	
Radiation therapy technique			
VMAT + IMRT	71 (100)	39 (27.1)	<0.0001
3D conformal	0	105 (72.9)	
Days to first durvalumab from completion of chemoradiation			
14–42 d	20 (28.2)	*NA*	
>42 d	51 (71.8)	*NA*	
Mediastinoscopy at diagnosis			
Not done	47 (66.2)	109 (75.7)	0.315
Negative	6 (8.5)	8 (5.6)	
Positive	18 (25.4)	27 (18.8)	
Brain MRI at diagnosis			
Not done	4 (5.6)	51 (35.4)	<0.0001
Done	67 (94.4)	93 (64.6)	
Death			
Death related to NSCLC	5 (7.0)	87 (60.4)	0.0048
Death unrelated to NSCLC	4 (5,6)	6 (4.2)	
Alive	62 (87.3)	51 (35.4)	

Abbreviation: ECOG, eastern cooperative oncology group; NOS, not otherwise specified; PDL1, programed cell death ligand 1; 3D, 3 dimensional; VMAT, volume metric arc therapy; IMRT, intensity modulated radiation therapy; d, days; MRI, magnetic resonance imaging; NSCLC, non‐small cell lung cancer.

### Treatments

#### Chemotherapy

All patients in the durvalumab group (*n* = 71, 100%) and almost all patients in the historical control group received concurrent chemoradiation (*n* = 135, 94%, *p* = 0.03). The majority of patients in both groups were treated with platinum‐based doublet chemotherapy using either carboplatin with paclitaxel (*n* = 145, 67.4%) or less frequently cisplatin with etoposide (*n* = 23, 10.7%). The remaining patients (*n* = 47, 21.9%) were treated using either single agent or other doublet platinum‐based chemotherapy. There was greater variation in the chemotherapy protocols used in the historical controls than the durvalumab cohort (*p* < 0.001). Durvalumab was used only after September 2017.

#### Radiation

Datasets for radiation therapy planning were obtained using a CT simulator (Phillips) in 3D mode for dose calculation and in 4D mode to assess respiratory motion. When feasible, motion management techniques such as deep inspiratory breath hold (DIBH) or continuous positive airway pressure (CPAP) were used to reduce lung and heart exposure during radiation treatments. Radiation treatments were planned with a computerized treatment planning system (Varian Eclipse). Radiation treatments were delivered on a linear accelerator (Varian) using conformal treatment planning techniques including 3D‐conformal, IMRT, or VMAT in all patients. All patients in the durvalumab group were planned with VMAT or IMRT (*n* = 71). The historical controls differed from the durvalumab group because only 39 patients were planned with VMAT or IMRT and the remaining 105 patients were planned with 3D‐conformal techniques (*p* < 0.001). Radiation therapy was delivered to a dose of 56–66 Gy in all patients.

### Survival

**FIGURE 1:**
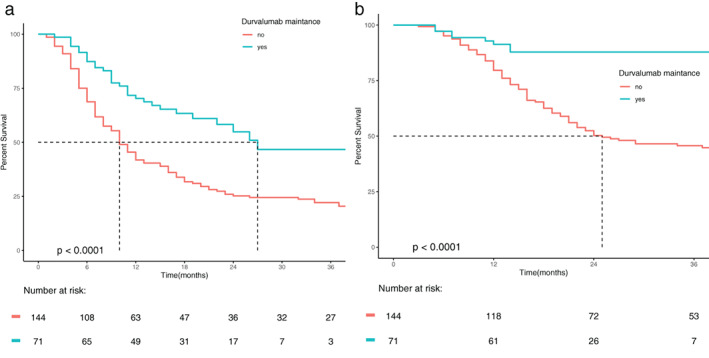
(a) KM showing progression free survival for durvalumab and historic controls. (b) KM showing overall survival for durvalumab and historic controls.

Figure 1(a),(b) shows that use of adjuvant durvalumab was associated with increased PFS and OS, median and 1‐year PFS with use of durvalumab were: 27 months and 83.1% versus 10 months and 43.8% for the historical controls (*p* < 0.0001). Median and 1‐year OS with use of durvalumab were not reached (NR) and 85.9% versus 24 months and 81.9%, (*p* < 0.0001) for the historical controls.

Table 2 shows the UVA and MVA evaluating patient characteristics and treatment parameters of the entire cohort correlation with PFS and OS. Univariate analysis showed weight loss was associated with decreased PFS (hazard ration [HR], 1.66; confidence interval [CI], 1.15–2.39; *p* = 0.006), stages IIIB and IIIC with decreased OS (HR, 1.89; 95% CI, 1.26–2.84; *p* = 0.002), use of IMRT or VMAT with improved PFS (HR, 0.63; CI, 0.45–0.89; *p* = 0.008), and OS (HR, 0.46; 95% CI, 0.30–0.72; *p* = 0.0006) and use of adjuvant durvalumab with increased PFS (HR, 0.44; 95% CI, 0.29–0.66; *p* < 0.0001) and OS (HR, 0.25; 95% CI, 0.13–0.51; *p* = 0.0001). Multivariate analysis showed treatment with adjuvant durvalumab was associated with increased PFS (HR, 0.44; 95% CI, 0.26–0.73; *p* = 0.001) and OS (HR, 0.31; 95% CI, 0.137–0.7; *p* = 0.005) and weight loss was associated with decreased PFS (HR, 1.59; 95% CI, 1.10–2.30; *p* = 0.015), but not with decreased OS.

**TABLE 2:** Univariate and multivariate analysis comparing variables for progression free survival and overall survival in the durvalumab and historical controls

Chemoradiation ± durvalumab		PFS				OS			
		UVA		MVA		UVA		MVA	
Parameter	*N* = 215	HR (95% CI)	*p*‐value	HR (95% CI)	*p*‐value	HR (95% CI)	*p*‐value	HR (95% CI)	*p*‐value
Gender									
Male	152	NR				NR			
Female	63	1.14 (0.80–1.63)	0.474			1.26 (0.82–1.94)	0.295		
Marital status									
Single	66	NR				NR			
Married	149	1.07	0.7			0.92 (0.61–1.41)	0.714		
Smoking status									
Never smoker	21	NR				NR			
Ever smoker	194	0.83 (0.49–1.43)	0.508			1.48 (0.69–3.19)	0.319		
Radiation therapy technique									
3D conformal	105	NR		NR		NR		NR	
VMAT + IMRT	110	0.63 (0.45–0.89)	0.008	1.11 (0.73–1.70)	0.615	0.46 (0.30–0.72)	0.0006	1.03 (0.59–1.78)	0.919
Weight loss									
No (<5%)	75	NR		NR		NR			
Yes (≥5%)	140	1.66 (1.15–2.39)	0.006	1.60 (1.11–2.30)	0.012	1.42 (0.92–2.19)	0.11		
Performance status									
ECOG 0	92	NR				NR		NR	
ECOG 1	107	1.21 (0.87–1.71)	0.254			1.33 (0.87–2.02)	0.1829	1.40 (0.89–2.19)	0.145
ECOG ≥2	16	1.17 (0.61–2.24)	0.631			1.88 (0.90–3.90)	0.09	1.69 (0.77–3.68)	0.188
Histology									
Adenocarcinoma	108	NR				NR			
Squamous cell carcinoma	71	1.07 (0.75–1.53)	0.699			1.16 (0.75–1.79)	0.508		
NOS	36	0.93 (0.58–1.48)	0.757			1.28 (0.75–2.18)	0.365		
Stage									
IIIA	100	NR				NR		NR	
IIIB–C	115	0.97 (0.71–1.35)	0.875			1.89 (1.26–2.84)	0.002	1.54 (1.00–2.37)	0.049
Mediastinoscopy at diagnosis									
Not done	156	NR				NR		NR	
Negative	14	0.64 (0.31–1.32)	0.226			0.33 (0.11–1.06)	0.0623	0.34 (0.10–1.12)	0.076
Positive	45	0.87 (0.58–1.32)	0.523			0.72 (0.42–1.21)	0.2102	0.96 (0.54–1.69)	0.879
Brain MRI at diagnosis									
Done	160	NR				NR		NR	
Not done	55	1.03 (0.72–1.47)	0.868			1.50 (0.99–2.26)	0.0507	1.05 (0.68–1.62)	0.838
Chemotherapy									
Carboplatin + paclitaxel	145	NR				NR		NR	
Cisplatin + etoposide	23	0.97 (0.55–1.71)	0.922			0.87 (0.41–1.82)	0.709	1.21 (0.55–2.64)	0.632
Other	47	1.23 (0.85–1.79)	0.27			1.76 (1.16–2.68)	0.008	1.31 (0.81–2.10)	0.274
Mode of chemo RT									
Sequential	9	NR				NR		NR	
Concomitant	206	0.80 (0.39–1.64)	0.542			0.47 (0.23–0.97)	0.042	0.88 (0.38–2.02)	0.756
Durvalumab treatment									
No	144	NR		NR		NR		NR	
Yes	71	0.44 (0.29–0.66)	<0.0001	0.42 (0.25–0.70)	<0.001	0.25 (0.13–0.51)	0.0001	0.30 (0.13–0.0.67)	0.003

### Immune‐related AE

**FIGURE 2:**
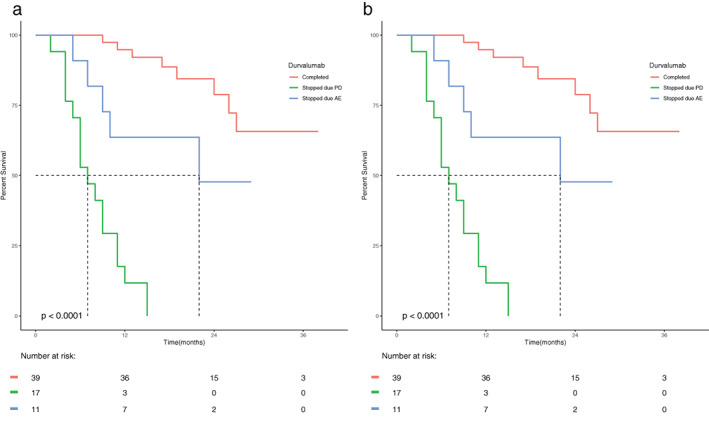
(a) KM showing effect of treatment interruption on PFS. (b) KM showing effect of treatment interruption on OS.

Immune‐mediated AE that occurred in the durvalumab group are summarized in Table 3. Most AE were grade 1–2 (*n* = 46, 65%). Grade 3–4 adverse events were uncommon (*n* = 6, 8.5%). Pneumonitis was the most common AE reported: low grade (*n* = 31, 43.7%), high grade (*n* = 4, 5.6%). There was one case of grade 3 encephalitis and one case of grade 3 esophagitis that occurred within a prior radiation field. All six patients who suffered a grade 3–4 AE remained free of progressive disease. There were no grade 5 AEs. Univariate analysis showed no association between PFS or OS and any grade AE, grade 3–4 AE, or pneumonitis. However, Figure 2(a),(b) shows that if an AE was associated with discontinuation of adjuvant durvalumab (*n* = 11), PFS and OS were decreased.

**TABLE 3:** Immunotherapy related adverse events

Adverse events							
	Immune‐related adverse events in the durvalumab group						
Toxicities (CTCAE grade)	No.	%	Grade 1–2	%	Grade 3–4	%	Comments
Patients with AEs reported	52	73.2	46	64.8	6	8.5	All 6 patients with a grade 3–4 adverse event remain free of PD
Total AEs reported	63	88.7	57	80.3	6	8.5	
Pneumonitis	35	49.3	31	43.7	4	5.6	
Hepatitis	3	4.2	3	4.2	0	0	
Nephritis	1	1.4	1	1.4	0	0	
Dermatologic	7	9.9	7	9.9	0	0	
Endocrine	10	14.1	10	14.1	0	0	Thyroiditis
Esophagitis	3	4.2	2	2.8	1	1.4	Radiation recall esophagitis
Colitis	1	1.4	1	1.4	0	0	
Encephalitis	1	1.4	0	0	1	1.4	
Arthritis	2	2.8	2	2.8	0		

Abbreviation: AE, adverse events; PD, progressive disease; CTCAE, common terminology criteria adverse events.

### Durvalumab subgroup

Most patients in the durvalumab group did not meet PACIFIC trial eligibility (*n* = 53, 75%) because of delay in initiation of adjuvant durvalumab beyond 42 days (*n* = 51, 72%), ECOG performance status ≥2 (*n* = 4, 6%), or prior history of immune disease (*n* = 3, 4%, sarcoidosis *n* = 2, and psoriasis *n* = 1). Four patients met two different PACIFIC trial exclusion criteria. Univariate analysis of the durvalumab group alone failed to identify any patient characteristics or treatment parameters associated with PFS or OS (Table S1).

PD‐L1 status was checked on archived samples from 51 patients in the durvalumab group. Archived samples were obtained in 47 patients before initiating chemoradiation, in 4 patients after completing chemoradiation, and in 2 patients on separate samples obtained both before and after chemoradiation. The presence or absence of PD‐L1 expression was not associated with PFS or OS (Table S1).

Thirty‐nine patients (55%) completed 1 year of adjuvant durvalumab without interruption. Durvalumab was discontinued in 32 patients (45%) because of: PD (*n* = 17, 24%), AE (*n* = 11, 15%), COVID‐19 pandemic (*n* = 3, 4%), or unrelated death (*n* = 1, 1%).

## DISCUSSION

The results of this single institution retrospective analysis demonstrate that for patients with locally advanced stage III NSCLC, the use of adjuvant durvalumab after completion of chemoradiation improves PFS and OS compared to historic controls treated with chemoradiation alone. Although most patients in our trial did not conform to eligibility criteria for inclusion in the PACIFIC trial because of delay in starting adjuvant durvalumab (*n* = 51), history of immune disease (*n* = 3), or ECOG status ≥2 (*n* = 4), the improved PFS and OS with adjuvant durvalumab observed in our study are consistent with outcomes reported in the PACIFIC trial and other real‐world studies.^7^, ^8^, ^9^, ^10^, ^11^, ^12^, ^13^, ^14^, ^15^, ^16^, ^17^, ^18^ The real‐world data presented in this study, although limited, suggest that future clinical trials consider inclusion of patients who begin durvalumab therapy beyond 42 days after completion of chemoradiation, have a prior history of immune disease, or have an ECOG performance status ≥2.

Univariate analysis of the durvalumab group showed that delay in starting durvalumab was not associated with decreased PFS or OS (Table S1). Although reasons for starting durvalumab beyond 42 days were not recorded, toxicity and slow recovery time following completion of chemoradiation therapy and logistics in completing imaging examinations after completing chemoradiation were probable causes for delay in starting adjuvant durvalumab. These results are consistent with other reports showing delay in starting adjuvant durvalumab beyond 42 days and do not adversely affect survival and that treatment delay beyond 42 days should not be considered a contraindication to initiating adjuvant durvalumab therapy.^12^ ^,^ ^15^ Antonia et al.,^8^ reporting on data from the PACIFIC trial suggested starting adjuvant durvalumab within 14 days of completion of chemoradiation may provide survival benefit compared with starting more than 14 days after completion of chemoradiation. However, the number of grade 3–4 AE and absence of grade 5 AE in our cohort was lower than the number of serious AE events reported in the PACIFIC trial. Potentially, the delay in commencing adjuvant durvalumab beyond 42 days may have contributed to the low rate of grade 3–4 AE reported in our cohort. Although, the optimal time for initiation of adjuvant durvalumab therapy to enhance survival and minimize risk of AE remains unknown, it may be prudent to avoid unnecessary delay and start therapy with adjuvant durvalumab as soon as possible after completing chemoradiation.

Three patients with immune disease were included in our cohort. One of these, a patient with psoriasis, suffered grade 3 encephalitis 6 months after completion of durvalumab, was treated with steroids and remains free of disease recurrence. Our limited data are consistent with data from Faehling et al.,^22^ who included nine patients with a history of autoimmune disease in their cohort and reported PFS and OS similar to patients without autoimmune disease, but with a greater rate of AE and suggested patients with history of autoimmune disease be considered for inclusion in future trials.

Several real‐world adjuvant durvalumab trials included small groups of patients with ECOG performance status ≥2 without identifying these patients at risk for worse outcomes or increased risk of AE.^12^, ^15^ Bi et al.^23^ showed no difference in treatment compliance, toxicities, or outcomes between patients with ECOG 2 and ECOG 0–1 performance scores receiving chemoradiation for stage III NSCLC. Two of four patients with ECOG performance scores ≥2 that were included in our durvalumab cohort remain alive and free of disease, suggesting adjuvant durvalumab may also benefit patients with ECOG performance scores ≥2. One trial evaluating concurrent radiotherapy with daily carboplatin followed by adjuvant durvalumab is currently accruing patients up to age 74 with ECOG performance status ≥2.^15^

It is unclear if patients without PD‐L1 receptors benefit from treatment with adjuvant durvalumab following chemoradiation. Paz‐Ares et al.^24^ in a post‐hoc analysis of PACIFIC trial data demonstrated improved OS and PFS in patients with PD‐L1 ≥1% compared to patients without PD‐L1 receptor expression. Desilets et al.^12^ also showed improved OS in patients with PD‐L1 ≥50%. In contrast, Faehling et al.^22^ showed PD‐L1 receptor status offered no difference in OS or DFS. Our data (Table S1) show both PFS and OS were unaffected by PD‐L1 expression that suggests PD‐L1 staining may not be useful for determining response to durvalumab. These data suggest that limiting use of durvalumab to patients with PD‐L1 receptor positivity may be unduly restrictive and that the absence of PD‐L1 receptors should not be considered an exclusion criterion for receiving treatment with adjuvant durvalumab until additional data is available.

We were unable to demonstrate an association between occurrence of an AE and OS or PFS. However, KM analysis suggests that interruption of durvalumab therapy because of an AE is associated with decreased OS and PFS (Figure 2(a),(b)). Desilets et al.^12^ has shown that development of pneumonitis is associated with decreased OS, but did not consider the effect of discontinuation of durvalumab treatment on OS. Our data suggest that discontinuation of durvalumab rather than the development of an AE may be the cause of diminished survival. This finding suggests caution should be exerted when considering discontinuation of adjuvant durvalumab for an AE. Further evaluation of outcomes following delay or discontinuation of adjuvant durvalumab is warranted.

Since the introduction of adjuvant durvalumab at our institution in 2017, almost all patients with stage III NSCLC are now offered tri‐modality with either surgery or adjuvant durvalumab following completion of definitive chemoradiation. The increased percentage of patients with stage IIIA disease in the durvalumab group compared to the historical controls (59.2% vs. 40.3%, *p* = 0.13) may suggest an increasing tendency to select adjuvant durvalumab rather than surgery as the third treatment modality for patients with stage III NSCLC. Further study of outcomes in patients receiving adjuvant durvalumab compared to surgery following chemoradiation is warranted. Furthermore, extending use of adjuvant durvalumab as a fourth treatment modality to patients who receive surgery remains to be considered as an additional therapeutic intervention.

The results of this study are limited because of the retrospective nature of the analysis, the absence of PD‐L1 data for the entire cohort, and the small number of patients in subgroups such as ECOG status ≥2 or with a history of prior immune disease. Compared to the historical controls, within the durvalumab group, there was greater use of MRI imaging for brain assessment, increased use of IMRT/VMAT, and an increased number of patients with stage IIIA disease. The increased percentage of patients with stage IIIA disease in the durvalumab cohort may have contributed to the more favorable outcomes in the durvalumab cohort because the presence of stage IIIB‐C disease was independently associated with worse OS on MVA (*p* = 0.049). Although single institution data may be limited because of institution bias, comparison to a cohort of historical matched controls treated from a single institution allows evaluation of effects of adjuvant therapy without confounders and variances observed in multicenter studies.

Strengths of this single institution study are its presentation of real‐world data to test reproducibility and provide confirmation of study results as well as provided anecdotal data useful for hypothesis generation and formulation of future controlled trials. For example, our data suggest that PD‐L1 receptor positivity may not be useful for predicting response to durvalumab and use of this as a selection criterion may exclude patients who may benefit from adjuvant durvalumab from receiving treatment.

## CONCLUSIONS

The results of this single institution retrospective analysis suggest that for patients with locally advanced stage III NSCLC, the use of adjuvant durvalumab after completion of chemoradiation improves PFS and OS compared to historic controls treated with chemoradiation alone even if elapsed time to initiation of durvalumab therapy was >42 days beyond completion of chemoradiation.

## CONFLICT OF INTEREST

J.B. reports grants from Merck Sharp and Dohme (MSD), AstraZeneca, Roche, Takeda, and AbbVie; consulting fees from MSD, AstraZeneca, Bristol Myers Squibb (BMS), Roche, Novartis, Causalis, AbbVie, and Takeda; honoraria for lectures from AbbVie, MSD, and AstraZeneca; and stock options from Causalis. He was the past chair of the Israel Lung Cancer Group. A.O. reports honoraria for lectures from Boehringer Ingelheim (BI), MSD, AstraZeneca, and Novartis. S.A. reports honoraria for lectures from MSD and AstraZeneca. D.U. reports consulting fees from MSD, BMS, BI, Roche, Takeda, and AstraZeneca. H.GS. reports honoraria for lectures from Roche, Genentech, MSD, AstraZeneca, Takeda, Astellas, BMS, Pfizer, and Medison.

## Supporting information


**Table S1** Univariate and multivariate analysis comparing variables for progression free survival and overall survival in the durvalumab subgroup.
