# Supplementary material for: Chemoradiation followed by adjuvant durvalumab in stage III non–small cell lung cancer: Real‐world comparison of treatment outcomes to historical controls treated with chemoradiation alone
Source: Thorac Cancer. 2022 May 11;13(12):1763–71. doi: 10.1111/1759-7714.14452 (PMC9200887; doi:10.1111/1759-7714.14452)
Supplement: Supplementary file 1 — Table S1 Univariate and multivariate analysis comparing variables for progression free survival and overall survival in the durvalumab subgroup. [file TCA-13-1763-s001.docx]

Table 1S: Univariate and multivariate analysis comparing variables for progression free survival and overall survival in the durvalumab subgroup.

| Durvalumab only  PD-L1 analysis |  |  |  |  |  |  |
| --- | --- | --- | --- | --- | --- | --- |
|  | Chemoradiation followed by durvalumab (n=71) | | UVA PFS |  | UVA OS |  |
| Parameter |  |  | HR (95% CI) | p value | HR (95% CI) | p value |
| ***Age*** | Median age (range) | 67 (40-82) | 0.98 (0.94-1.02) | 0.31 | 1.03(0.95-1.12) | 0.419 |
| ***Gender*** | Male | 45 (63.4) | NR |  | NR |  |
|  | Female | 26 (36.6) | 1.01 (0.47-2.16) | 0.975 | 1.6 (0.43-5.97) | 0.488 |
| ***Marital Status*** | Single | 23 (32.4) | NR |  | NR |  |
|  | Married | 48 (67.6) | 2.08 (0.85-5.11) | 0.108 | 3.82 (0.48-30.6) | 0.207 |
| ***Performance status*** | ECOG 0 | 34 (47.9) | NR |  | NR |  |
|  | ECOG 1 | 33 (46.5) | 1.53 (0.71-3.27) | 0.277 | 1.37 (0.31-6.13) | 0.679 |
|  | ECOG ≥2 | 4 (5.6) | 2.02 (0.44-9.15) | 0.364 | 3.25 (0.34-31.27) | 0.309 |
| ***Smoking status*** | Ever Smoker | 64 (90.1) | 1.11 (0.34-3.66) | 0.866 | NA |  |
|  | Never Smoker | 7 (9.6) | NR |  | NA |  |
| ***Weight loss*** | yes (>5%) | 43 (60.6) | 2.74 (1.12-6.70) | 0.028 | 1.05 (0.25-4.39) | 0.949 |
|  | no (<5%) | 28 (39.4) | NR |  | NR |  |
| ***Histology*** | Adenocarcinoma | 39 (54.9) | NR |  | NR |  |
|  | Squamous Cell Carcinoma | 23 (32.4) | 1.58 (0.75-3.33) | 0.23 | 1.23 (0.32-4.78) | 0.302 |
|  | NOS | 9 (12.7) | 0.47 (0.11-2.07) | 0.319 | NA | NA |
| ***Stage*** | IIIA | 42 (59.2) | NR |  | NR |  |
|  | IIIB-C | 29 (40.8) | 0.57 )0.25-1.27) | 0.168 | 0.51 (0.10-2.51) | 0.404 |
| ***Prior autoimmune disease*** | Yes | 3 (4.2) | 0.56 (0.08-4.15) | 0 | NA |  |
|  | No | 68 (95.8) | NR | 0.574 | NA |  |
| ***PDL1 status**** | ≥50% | 20 (28.2) | 1.53 (0.68-3.42) | 0.303 | 0.76 (0.14-4.14) | 0.751 |
|  | ≥1-49% | 9 (12.7) | 0.63 (0.17-2.31) | 0.486 | 0.56 (0.05-5.89) | 0.632 |
|  | <1% | 22 (31.0) | NR |  | NR |  |
| ***PDL1 status*** | ≥1% | 29 (40.8) |  |  | 0.70 (0.14-3.48) | 0.664 |
|  | <1% | 22 (31.0) |  |  | NR |  |
| ***Chemotherapy*** | Carboplatin + Paclitaxel | 54 (76.1) | NR |  | NR |  |
|  | Cisplatin + Etoposide | 14 (19.7) | 1.05 (0.43-2.58) | 0.915 | 0.5 (0.06-4.11) | 0.524 |
|  | Other | 3 (4.2) | 0.71 (0.10-5.28) | 0.74 | NA |  |
| ***Mode of chemoradiation*** | Sequential | 0 | NA |  | NA |  |
|  | Concomitant | 71 (100) | NA |  | NA |  |
| ***Radiation Therapy Technique*** | VMAT+IMRT | 71 (100) | NA |  | NA |  |
|  | 3D conformal | 0 | NA |  | NA |  |
| ***Days to first durvalumab from completion of chemoradiation*** | 14-42 days | 20 (28.2) | NR |  | NR |  |
|  | > 42 days | 51 (71.8) | 1.0 (0.99-1.01 | 0.926 | 0.99 (0.97-1.02) | 0.675 |
| ***Mediastinoscopy at diagnosis*** | not done | 47 (66.2) | NR |  | NR |  |
|  | negative | 6 (8.5) | 2.73 (1.02-7.30) | 0.045 | 5.86 (1.27-26.97) | 0.023 |
|  | positive | 18 (25.4) | 0.54 (0.20-1.46) | 0.226 | 1.41 (0.26-7.80) | 0.691 |
| ***Brain MRI at diagnosis*** | not done | 4 (5.6) | 1.32 (0.31-5.62) | 0.704 | NA |  |
|  | done | 67 (94.4) | NR |  | NA |  |
| ***Death*** | Death related to NSCLC | 5 (7.0) |  |  |  |  |
|  | Death unrelated to NSCLC | 4 (5,6) |  |  |  |  |
|  | alive | 62 (87.3) |  |  |  |  |
| ***Overall survival*** | 1-year OS | 91% (0.85-0.98) |  |  |  |  |
|  | Median 0S in months (range) | NR (38-NR) |  |  |  |  |
| ***Progression free survival*** | 1-year PFS | 71% (60-82) |  |  |  |  |
